# A Much Underpaid Profession

**Published:** 1920-03-06

**Authors:** 


*To the Editor of* The Hospital.

### A Much Underpaid Profession.

*To the Editor of* The Hospital.

Sir,—In reference to Guardian's letter in your issue of February 28, I agree that nursing salaries in many instances need reconsideration on liberal lines. But nothing is gained by stating their case unfairly. Throughout his letter your correspondent makes no mention whatever of the fact that the nurses in question are boarded, lodged, to some extent clothed, and have their laundry expenses paid. Every rise in the cost of these emoluments means an increase in salary, for the greater part of the " bare necessities of life " are paid for in cash by the employer.

While on the subject of women's salaries, may I remark that it seems curious to a mere man that a body like the College of Nursing, which advocates fair play for nurses, can offer only £200 a year as the salary of an organising secretary, an important officer, who will have to feed, house, clothe herself, pay for medical attendance, holidays, and recreations out of a wage which is less than that demanded by a dock labourer?

